# *Acanthamoeba* spp. in Dialysis Water: Assessing the Potential Risk of Transmission to Hemodialysis Patients

**DOI:** 10.1007/s11686-025-00992-6

**Published:** 2025-02-07

**Authors:** Mona Mohamed Tolba, Heba Elhadad, Shaban Hassan Abu Kabsha, Nesma Seyam El-Kady, Safia Saleh Khalil, Amira Hussein Mohamed, Hend Aly El-Taweel

**Affiliations:** 1 Department of Parasitology, Medical Research Institute, Alexandria University, 165 El-Horeya Rd, Al Ibrahimeyah Qebli WA Al Hadrah Bahri, Alexandria, Egypt; 2 Behera Water and Drainage Company, Damanhour, Egypt; 3 Department of Experimental and Clinical Internal Medicine, Medical Research Institute, Alexandria University, Alexandria, Egypt

**Keywords:** *Acanthamoeba* spp., Water, Dialysis, Anti-*Acanthamoeba* IgG

## Abstract

**Purpose:**

*Acanthamoeba* spp. can colonize various freshwater habitats. They are the causative agents of granulomatous amoebic encephalitis and can harbor many microorganisms. We studied the presence of *Acanthamoeba* spp. in the water system of a hemodialysis unit and assessed the potential for transmission to hemodialysis patients.

**Methods:**

Water samples collected from pretreatment and posttreatment water of the treatment station and from input and output water of the dialysis machines were cultured on non-nutrient agar supplemented with *Escherichia coli*. Blood samples from dialysis patients in the unit and from a control group in the same hospital were tested for anti-*Acanthamoeba* IgG antibodies.

**Results:**

*Acanthamoeba* spp. were found in posttreatment water. They were more commonly found in the input water samples (79.2%), than in the output water samples (16.7%) (*p* = 0.001). Anti-*Acanthamoeba* IgG antibodies were present in 32.9% of patients and were absent in the control group (*p* = 0.002). The duration of hemodialysis was significantly longer in seropositive patients than in seronegative patients (*p* = 0.008).

**Conclusion:**

The study highlights the presence of *Acanthamoeba* spp. in the dialysis system. The relatively high prevalence of anti-*Acanthamoeba* IgG antibodies and the link between dialysis duration and seropositivity emphasize the importance of rigorous water monitoring.

## Introduction

*Acanthamoeba* spp. are free-living amoebae (FLA) known to cause fatal granulomatous amoebic encephalitis in immunocompromised patients and *Acanthamoeba* keratitis in contact lens wearers [1]. They also cause sinusitis and cutaneous or disseminated lesions in AIDS patients [2]. Furthermore, *Acanthamoeba* can serve as a potential reservoir for several waterborne pathogens, protecting them against adverse environmental conditions, host defense mechanisms and therapeutic agents [3].

*Acanthamoeba* can be found in various natural environmental sources, such as soil, dust and different water habitats [4, 5]. In water reservoirs, they are frequently associated with biofilms, which serve as a means of protection and dissemination for *Acanthamoeba* spp [6]. Contamination by *Acanthamoeba* species has been detected in various water systems in hospitals, potentially endangering patient health [7–9].

This amoeba has two stages: the trophozoite and the cyst. Trophozoites undergo binary fission and flourish in water in the presence of a food source and other suitable physical conditions, such as temperature, pH and osmolarity. Cysts, which have a protective double wall, are formed under adverse environmental conditions [10–12]. Cysts of *Acanthamoeba* are resistant to alcohol and aldehyde-based disinfectants, which are often used for the disinfection of medical equipment. Chlorine applied at low residual concentrations of 2 to 5 ppm in drinking water treatment is ineffective against *Acanthamoeba* [13, 14].

In patients with end-stage renal disease, infectious diseases are frequent and represent a leading cause of mortality. Elevated uremia is associated with immune dysfunction, making these patients especially prone to infection. Dialysis procedures further increase the chances of exposure to pathogenic microorganisms. Insufficient disinfection and maintenance of the water treatment and distribution system contribute to dialysis-related infections [15]. Microorganisms can survive and grow in dialysate fluid [16]. Furthermore, pathogens can multiply in water residues present inside pipes and storage tanks, forming biofilms that potentially help protect and disseminate *Acanthamoeba* spp [6].

The dialysis units in hospitals are equipped with water treatment units and a hydraulic system to distribute the purified water. The treatment process involves the utilization of filters with varying pore sizes, reverse osmosis and disinfection [17]. The treated water is then mixed with the dialysis concentrate in dialysis machines to produce dialysate fluid with the desired concentration of elements [18]. There have been several reports of *Acanthamoeba* spp. detection in the hydraulic system of dialysis units, with rates ranging from 17 to 40% [19–22]. However, the clinical significance of these findings remains unclear.

The present work investigated the presence of *Acanthamoeba* spp. in water samples collected at different points in a hemodialysis unit and evaluated the potential for transmission to hemodialysis patients.

## Materials and Methods

### Study Setting

The study was conducted in the dialysis unit of the Medical Research Institute, Alexandria University, from October 2021 to January 2022. The unit contains 24 dialysis machines and is equipped with a pretreatment station to filter and disinfect drinking water supplied by the main water treatment plant in Alexandria. The treated water is then delivered through a distribution system to 24 dialysis machines where the dialysate is produced and carried to the dialyzer.

The study protocol was presented to the Research Ethics Committee of the Medical Research Institute, Alexandria University (IORG0008812), for approval before the beginning of the study (serial number: E/C. S/N, 86/2021). All procedures were carried out in compliance with relevant laws and guidelines, and with the ethical standards of the Declaration of Helsinki. The committee was constructed and operated according to ICH GCP guidelines and applicable local and institutional regulations and guidelines. Oral informed consent was obtained from each participant prior to their inclusion in the study.

### Collection of Water Samples

A total of 52 water samples, one liter each, were collected, including two pretreatment and two posttreatment water samples from the main water treatment station and one input and one output sample from each of the 24 dialysis machines in the Dialysis Unit. Samples were collected in prelabeled autoclavable polypropylene stoppered containers. The collected water samples were immediately filtered through nitrocellulose membrane filters (0.8 μm pore size).

### Acanthamoeba Culture

The filters were inverted face to face on the surface of non-nutrient agar medium seeded with living *E. coli* bacteria and incubated at 37 °C for one week. The cultures were subjected to daily microscopic examination using an inverted microscope for detection of *Acanthamoeba* spp [23]. Trophozoites of *Acanthamoeba* species were identified by the presence of acanthopodia, the characteristic locomotive pseudopodia [10]. Plates containing trophozoites of *Acanthamoeba* were left at room temperature for a few days and subjected to daily microscopic examination for cyst formation. Trophozoites and cysts were picked and placed on clean glass slides, fixed with methanol and stained with Giemsa stain.

### Human Samples

All hemodialysis patients attending the dialysis unit during the study period (*n* = 70) were included in the study. The patients’ ages ranged from 25 to 70 years, and 57% of them were males. The patients were undergoing three dialysis sessions per week and had been on maintenance hemodialysis for a duration ranging from one month to 28 years (mean = 8.22 ± 6.97 years). None of the patients were receiving immunosuppressive treatment. Additionally, a comparison group of 20 individuals [11 males and 9 females) within the same age range and working in the same hospital was included in the study.

A predesigned questionnaire was used to record demographic data, duration of dialysis medical history and clinical findings of patients. A single sample (approximately 3 ml) was collected from each participant by aseptic procedures in a dry, clean test tube prelabeled with the participant number. The samples were centrifuged (3000 rpm) for 5 min to obtain sera, which were stored in clean, labeled Eppendorf tubes at -20 °C for serological testing.

### Detection of Anti-*Acanthamoeba* Antibodies

Serum samples from participants were tested for anti-*Acanthamoeba* IgG antibodies using a qualitative enzyme immunoassay kit (Bioassay Technology Laboratory BT LAB, China Cat. NO ED0593Hu) according to the manufacturer’s instructions. The optical density of each sample was compared to that of the positive and negative controls to determine the presence of anti-*Acanthamoeba* IgG.

### Statistical Analysis

The data were analyzed using the IBM SPSS software package, version 20.0 (Armonk, NY: IBM Corp.). Comparisons of frequencies among groups were performed using the chi-squared test. Fisher’s exact p value was used if there was at least one cell with an expected frequency of < 5. The odds ratio (OR) and 95% confidence interval (CI) were calculated to determine the ratio of the odds of an event occurring in a group exposed to a risk factor to the odds in the non-exposed group. The Mann‒Whitney test was used to compare the duration of dialysis between seropositive and seronegative patients. A p value less than 0.05 was considered to indicate statistical significance.

## Results

### *Acanthamoeba* spp. in Water Samples

*Acanthamoeba* spp. were not detected in the pretreatment samples but were detected in the two posttreatment samples from the main water treatment station of the dialysis unit. In water samples from the dialysis machines, *Acanthamoeba* spp. were detected in 19 out of 24 input samples (79.2%) and 4 out of 24 output samples (16.7%), with a removal percentage of 78.9%. and a highly statistically significant difference (*p* < 0.001) (Table 1).

**Table 1:** *Acanthamoeba* spp. in input and output water samples of the dialysis machines

*Acanthamoeba* spp.	Input water (*n* = 24)		Output water (*n* = 24)		χ2	*P* ^1)^	Removal percent^2)^
	No.	%	No.	%			
Positive	19	79.2	4	16.7	18.783^*^	< 0.001^*^	15/19 = 78.9%
Negative	5	20.8	20	83.3			

^1)^: *P*-value < 0.05 was considered statistically significant

^2)^: Removal % = number of positive input samples that turned negative in the output/total number of positive input samples

χ^2^: Chi-square test

**Fig. 1:**
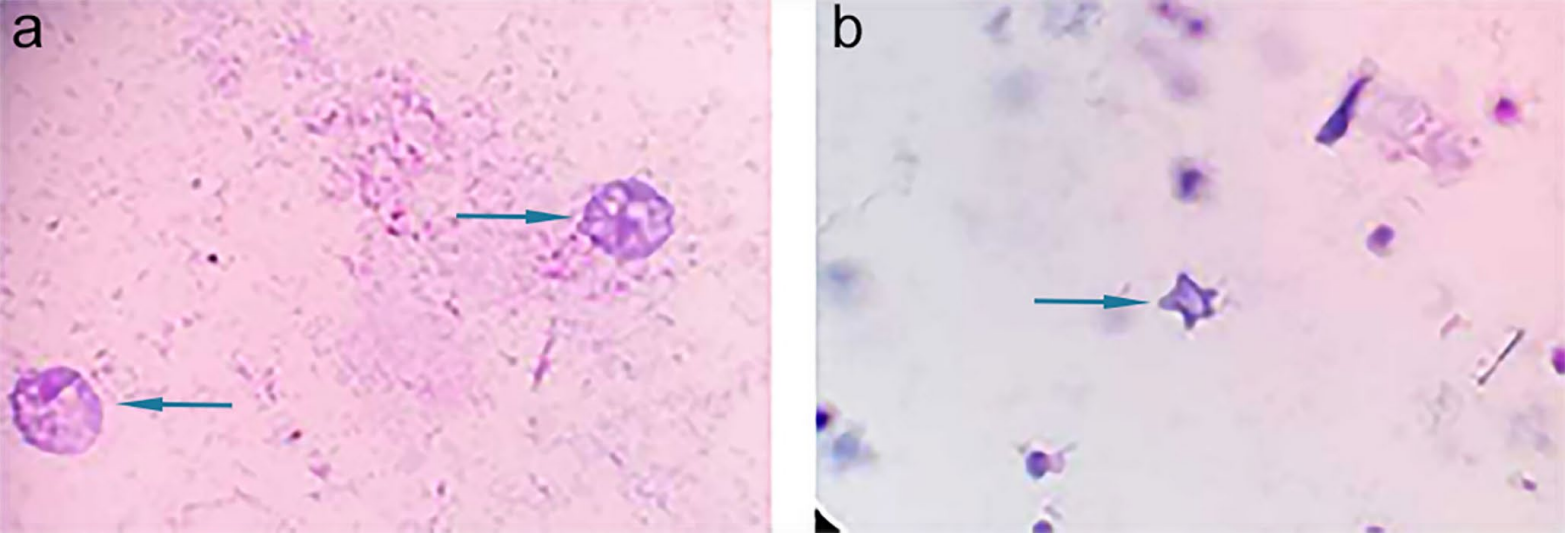
Giemsa-stained *Acanthamoeba* spp. in water samples from dialysis machines after cultivation in non-nutrient agar seeded with *E. coli* (**a**): trophozoites, (**b**): star shaped cyst (100× oil immersion lens)

In culture, *Acanthamoeba* trophozoites appeared as pleomorphic uninucleate structures measuring 15–45 μm. Some trophozoites showed acanthopodia as spiny surface projections. *Acanthamoeba* cysts appeared as double-walled, spherical, polygonal or star-shaped forms measuring 15–25 μm in diameter (Fig. 1).

### *Acanthamoeba* IgG Antibodies in Blood Samples

*Acanthamoeba* IgG antibodies were detected in 23 out of 70 hemodialysis patients (32.9%) and in none of the healthy controls (*p* = 0.002) (Table 2). The duration of maintenance hemodialysis was significantly longer in seropositive patients (median: 13 years, range: 3 months-28 years) than in seronegative patients (median: 4 years, range: one month to 22 years) (*p* = 0.008) (Table 3).

**Table 2:** Seroprevalence of *Acanthamoeba* IgG antibodies in the studied hemodialysis patients and controls

*Acanthamoeba* Antibodies	Hemodialysis patients (*n* = 70)		Controls (*n* = 22)		χ2	*P* ^1)^
	No.	%	No.	%		
Positive	23	32.9	0	0.0	9.638	0.002
Negative	47	67.1	22	100.0		

^1)^: *P*-value < 0.05 was considered statistically significant

χ^2^: Chi-square test

**Table 3:** *Acanthamoeba* seropositivity in hemodialysis patients according to the duration of hemodialysis

Duration of hemodialysis	Acanthamoeba antibodies in serum		U	*P* ^1)^
	Positive (*n* = 23)	Negative (*n* = 47)		
Median (Min.– Max.)	13.0 (0.25–28.0)	4.0 (0.08–22.0)	330.50	0.008

^1)^: *P*-value < 0.05 was considered statistically significant

U: Mann‒Whitney test

### Association of *Acanthamoeba* Seropositivity with Water Contamination

The 19 machines with positive input water were used by 55 patients, whereas the five machines with negative input water were used by 15 patients. Thirteen patients used four machines with positive-output water, and 57 patients used 20 machines with negative-output water. There was no significant association between the detection of *Acanthamoeba* spp. in the input or output water of the dialysis machines and the presence of anti-*Acanthamoeba* antibodies in hemodialysis patients (Table 4).

**Table 4:** Relationships between the presence of *Acanthamoeba* in the input and output water of the dialysis machine and seropositivity in hemodialysis patients

Water	No. of patients	Acanthamoeba Antibodies				OR (LL–UL 95% C.I.)	*P* ^1)^
		Positive (*n* = 23)		Negative (*n* = 47)			
		No.	%	No.	%		
Input water							
Positive	55	16	29.1	39	70.9	0.469 (0.146–1.510)	0.204
Negative^®^	15	7	46.7	8	53.3	1.000	
Output water							
Positive	13	1	7.7	12	92.3	0.133 (0.016–1.092)	0.060
Negative^®^	57	22	38.6	35	61.4	1.000	

^1)^: *P*-values < 0.05 were considered statistically significant

^®^: reference group, OR: odds ratio, CI: confidence interval, LL: lower limit, UL: upper limit

### Association of *Acanthamoeba* Seropositivity with Demographic and Medical Variables

There was no significant difference between the mean age of seropositive and seronegative patients (49.51 ± 13.34 and 50.61 ± 9.82 years, respectively; *p* = 0.699). There was no significant association between *Acanthamoeba* seropositivity and patient sex (*p* = 0.941), anemia (^FE^*p*=0.593), recurrent headache (^FE^*p*=1), hypertension (*p* = 0.673), diabetes mellitus (^FE^*p*= 1) or HCV infection (^FE^*p*=1) among the hemodialysis patients. *Acanthamoeba* antibodies were not detected in systemic lupus patients, but 17% of the seronegative patients had systemic lupus (*p* = 0.05) (Table 5).

**Table 5:** Association of *Acanthamoeba* seropositivity in hemodialysis patients with demographic and clinical variables

Variable	Acanthamoeba antibodies in serum		Test of significance	*P* ^1)^
	Positive (*n* = 23)	Negative (*n* = 47)		
Demographic data				
Age in years: Mean ± SD	50.61 ± 9.82	49.51 ± 13.34	t = 0.389	0.699
Gender: No. (%)				
Male	13 (56.5)	27 (57.4)	χ2 = 0.005	0.941
Female	10 (43.5)	20 (42.6)		
Medical data: No. (%)				
Recurrent headache	0(0.0)	2(4.3)	-	^FE^*p*=1.000
Anemia	21(91.3)	45(95.7)	-	^FE^*p*=0.593
Hypertension	11 (47.8)	25 (53.2)	χ2 = 0.178	0.673
Diabetes	3(13.0)	6 (12.8)	-	^FE^*p*=1.000
Hepatitis C	2 (8.7)	3 (6.4)	-	^FE^*p*=1.000
Systemic lupus	0 (0.0)	8 (17.02)	-	^FE^*p*=0.05

^1)^: *P*-values < 0.05 were considered statistically significant

t: t test; χ^2^: Chi-square test; FE: Fisher’s exact

## Discussion

Contamination of water in different hospital units with *Acanthamoeba* spp. carries a potential health risk to patients with impaired immunity [20, 24]. The results of the present study revealed that *Acanthamoeba* spp. were detected in posttreatment water from the main water treatment station and in most input water samples collected before entry to the dialysis machines, although they could not be found in pretreatment water samples. Pretreated water represents tap water supplied by the Drinking Water Treatment Plant (DWTP) in Alexandria. The absence of *Acanthamoeba* or contamination rates as low as 7% were reported in several recent studies in different Egyptian governorates [25, 26]. Generally, the prevalence of free-living amoebae in tap water is influenced by water turbidity, the study season and the quality of the applied water treatment method [27, 28]. However, a shift in microbial community structure, including that of *Acanthamoeba* spp., from treated water to point-of-use water may occur due to several factors, such as an intermittent water supply, residual chlorine, and prolonged storage [29].

Treatment procedures in the study unit involved the use of filters with gradually decreasing pore sizes to remove particles larger than 5 μm; a carbon filter to adsorb chlorine, chloramine, and other organic contaminants; a water softener to remove calcium and magnesium salts; and RO membranes that excluded more than 90% of the contaminants. Water was then passed through a nitrite filter with a pore size of 1 μm, an ultraviolet ray unit, and a filter with a pore size of 0.2 μm. These water treatment procedures collectively ensure adequate removal of contaminants, including *Acanthamoeba* trophozoites and cysts (size > 13 μm). Water quality is continuously monitored in the dialysis unit and checked regularly by the Ministry of Health. The detection of *Acanthamoeba* in posttreatment water samples despite the application of rigorous treatment steps may be attributed to biofilm formation in the distribution system. The biofilm adheres to solid surfaces and provides favorable conditions for the growth and proliferation of *Acanthamoeba* [6]. Biofilm formation and colonization by *Acanthamoeba* have been previously demonstrated in various water systems in hospitals [30, 31]. The accumulation of *Acanthamoeba* spp. in biofilms is enhanced by chlorine removal, which is required before mixing water with the dialysate fluid. The resistance of *Acanthamoeba* cysts to changes in temperature and pH and exposure to disinfectants promotes their survival [12].

In the present study, the detection rate of *Acanthamoeba* spp. was significantly lower in the output samples than in the input samples from the dialysis machine, with a removal percentage of 78.9%. Biofilm formation and *Acanthamoeba* contamination inside dialysis machines depend on the temperature of the dialysate, the levels of electrolytes and glucose in the fluid, and the existence of stagnant points in the machine’s circuit [32]. To minimize the presence of contaminants, dialysis machines often incorporate a final filtration system composed of microfilters and ultrafilters to ensure the removal of microorganisms and endotoxins. These filters have tiny pores, typically ranging from 0.1 to 2 μm, to sieve and eliminate contaminants. Moreover, the use of a diasafe filter in each machine produces ultrapure water for dialysis. The pore size of this filter is approximately 0.1 μm, which is less than the diameter of the parasite, so it prevents the passage of *Acanthamoeba* to the output water [15, 33].

An association between hemodialysis and *Acanthamoeba* seropositivity was demonstrated in the present study. *Acanthamoeba* IgG antibodies were detected in approximately one-third of the hemodialysis patients but none of the healthy controls. Moreover, the duration of maintenance hemodialysis was significantly longer in seropositive patients than in seronegative patients, with no relation to the cause of renal failure. However, the elevated risk of *Acanthamoeba* seropositivity in hemodialysis patients could not be linked to the presence of *Acanthamoeba* spp. in the water of the dialysis machines. This can be explained by several factors: (I) Dialysis occurs through a semipermeable membrane that separates the patient’s blood from the dialysate. Living *Acanthamoeba* spp. are too large to pass through this membrane. (II) IgG seropositivity represents cumulative exposure to *Acanthamoeba* or its antigenic fragments in repeated hemodialysis sessions rather than the current exposure to culturable amoebae.

The association between *Acanthamoeba* seropositivity and hemodialysis observed in the present study can be attributed to immunosuppression in patients with end-stage renal disease, which increases susceptibility to infection from other environmental sources.

In the present study, the frequency of *Acanthamoeba* seropositivity in hemodialysis patients did not significantly differ according to age or sex and was not related to any clinical manifestations in dialysis patients. Similar results were reported previously [34, 35]. Despite its ubiquitous nature, *Acanthamoeba*-related disease is relatively rare. Cases of granulomatous amoebic encephalitis have not been previously reported in Egypt. *Acanthamoeba* spp. are considered opportunistic pathogens of low virulence [36]. Pathogenicity has been linked to infection by T4 and certain other genotypes [12, 37] These genotypes represent a relatively small proportion of *Acanthamoeba* spp. detected in environmental samples [26]. Nevertheless, it is believed that many cases of *Acanthamoeba* infections may be unrecognized due to the nonspecific nature of symptoms and unavailability of diagnostic tools [38].

In conclusion, the present study demonstrated the presence of *Acanthamoeba* spp. in the dialysis system, emphasizing the importance of rigorous water quality monitoring and adherence to infection control measures in dialysis facilities. The relatively high prevalence of anti-*Acanthamoeba* IgG antibodies in hemodialysis patients indicates alarming exposure to *Acanthamoeba*. Although there was no significant association between the presence of *Acanthamoeba* in water samples and the seropositivity rate, the significant difference in the duration of dialysis between seropositive and seronegative patients underscores the need to investigate the clinical consequences of *Acanthamoeba* exposure in such vulnerable populations.

## Data Availability

The data that support the findings of this study are available from the corresponding author upon reasonable request.
